# The Standing of the Profession

**Published:** 1911-04-01

**Authors:** 


### THE STANDING OF THE PROFESSION.

A few weeks ago a lament was voiced in these columns over the misrepresentations and slanders with regard to the medical profession which are only too prevalent among certain sections of the public. At the same time an appeal was made for the adoption of more militant tactics in meeting the forces of prejudice and ignorance; for an offensive-defensive strategy in place of the calm waiting-in-entrenchments system. Thus, ran our argument, and thus only, can the whole duty of the profession to the public be fulfilled. It is interesting to notice that such comment as has been offered in the public press on this view, which is still unaltered, tends rather in the direction of denying the evils alluded to than in that of criticising the remedy proposed. The *Westminster Gazette*, for example, would have us believe that things are less black than we painted them, and that in reality the confidence of the nation in its doctors—more especially in its general practitioners—remains unshaken.

Of all poses, that of the misunderstood is perhaps one of the most obnoxious; and is at all hazards to be avoided. To convince our contemporary that there is much substance and no pose in the complaints we made, it may be well to compare the position of medicine with that of some of the other learned professions—premising that the modern developments of preventive medicine have indisputably advanced our science to a position in which its value to the body politic is greater far than it ever has been in times past. The House of Lords, for instance, contains two doctors, one of whom reached that somewhat precarious eminence not because he had been a professor of medicine, but because he subsequently took up politics and became a Minister. There are in fact more journalists and far more lawyers than doctors in that chamber, as every newspaper man knows. Then again, in municipal affairs the Medical Officer of Health ranks after the Surveyor. At Court the King's medical advisers yield precedence to his grooms of the chamber and masters of the horse, who are not necessarily Peers. To take the latest example from current events, the vogue of the "bonesetter" shows how ready people are—even those whose educational advantages should enable them to judge more shrewdly—to flock to irregular practitioners, notwithstanding the disastrous consequences which not so very rarely ensue. There is one highly respected surgeon in London who declares he has himself amputated nine or ten limbs in which the embers of tuberculous disease have been fanned into a blaze by the ministrations of one bonesetter alone out of the horde who flourish in England.

It is gratifying to read in the correspondence columns of the papers that there are still millions who respect the profession and admire its work. It was never our intention to contend otherwise. But those who write and feel this have no idea, as already explained in our previous article, how maligned and ridiculed the profession is in various quarters. Moreover, it is these latter sections of the populace that are most vocal; their leather-lunged invective penetrates many a humble home. The more insidious but equally false doctrines of the Christian Scientist and of the vast crowd of cranks and faddists who have peculiar theories of their own, do equal damage among the more affluent classes. It is to combat the delusions of these people, not to preach truth to those who are already believers, that a more energetic campaign against error is advocated.

